# Does screening for and intervening with multiple health compromising behaviours and mental health disorders amongst young people attending primary care improve health outcomes? A systematic review

**DOI:** 10.1186/s12875-016-0504-1

**Published:** 2016-08-04

**Authors:** Marianne J. Webb, Sylvia D. Kauer, Elizabeth M. Ozer, Dagmar M. Haller, Lena A. Sanci

**Affiliations:** 1 Department of General Practice, University of Melbourne, 200 Berkeley Street, Carlton, 3053 Australia; 2 Division of Adolescent & Young Adult Medicine, Department of Pediatrics, UCSF Benioff Children’s Hospital, University of California San Francisco, San Francisco, 94118 USA; 3 UCSF Office of Diversity and Outreach, University of California San Francisco, San Francisco, 94118 USA; 4 Primary Care Unit, Faculty of Medicine, University of Geneva, 9 av de Champel, 1211 Geneva 4, Switzerland; 5 Adolescent and Young Adult Program, Department of Community, Primary Care and Emergency Medicine and Department of Pediatrics, Geneva University Hospitals, 87 bvd de la Cluse, Geneva, 1205 Switzerland

**Keywords:** Adolescent, Screening, Primary care, Health compromising behaviour, Mental health, Prevention

## Abstract

**Background:**

Adolescence and young adulthood are important developmental periods. Screening for health compromising behaviours and mental health disorders during routine primary care visits has the potential to assist clinicians to identify areas of concern and provide appropriate interventions. The objective of this systematic review is to investigate whether screening and subsequent interventions for multiple health compromising behaviours and mental health disorders in primary care settings improves the health outcomes of young people.

**Methods:**

Using the Preferred Reporting Items for Systematic Reviews and Meta-Analyses (PRISMA) guidelines, literature searches were conducted in Medline, PsycINFO, Scopus and Cochrane Library databases (Prospero registration number CRD42013005828) using search terms representing four thematic concepts: primary care, young people, screening, and mental health and health compromising behaviour. To be eligible for inclusion, studies had to: include a measure of health outcome; include at least 75 % of participants aged under 25 years; use a screening tool that assessed more than one health domain; and be conducted within a primary care setting. Risk of bias was assessed using the Quality Rating Scale.

**Results:**

From 5051 articles identified, nine studies fulfilled the inclusion criteria and were reviewed: two randomised controlled trials (RCTs), one pilot RCT, two clustered RCTs, one randomised study with multiple intervention groups and no control group, one cluster RCT with two active arms, one longitudinal study and one pre-post study. Seven studies, including two RCTs and one clustered RCT, found positive changes in substance use, diet, sexual health or risky sexual behaviour, alcohol-related risky behaviour, social stress, stress management, helmet use, sleep and exercise. Of only two studies reporting on harms, one reported a negative health outcome of increased alcohol use.

**Conclusions:**

There is some evidence that the use of screening and intervention with young people for mental health disorder or health compromising behaviours in clinical settings improves health outcomes. Along with other evidence that young people value discussions of health risks with their providers, these discussions should be part of the routine primary care of young people. Further quality studies are needed to strengthen this evidence.

**Electronic supplementary material:**

The online version of this article (doi:10.1186/s12875-016-0504-1) contains supplementary material, which is available to authorized users.

## Background

### Health compromising behaviours and mental health in adolescence

Adolescence is an important developmental period for physical, cognitive, social and emotional growth. It is also a time when health and lifestyle habits and behaviours that influence adult health are initiated [1]. The terms adolescent, youth, teenager and young people are defined by different age ranges but also tend to be used interchangeably. A comprehensive overview of adolescent development presents a contemporary argument in favour of grouping these terms because the age range of 10 to 24 years captures the physical and psychosocial changes that typically occur in transition from childhood to adult maturity [1]. Hence, unless otherwise stated, the term *young people* is used in this article to refer to those aged 10 to 24.

In young people aged 10 to 24 substance use, poor diet, lack of exercise and mental disorders are the causes of significant disability [2]. Seventy-five per cent of mental illnesses begin before the age of 25 years with one in four young people experiencing mental health disorders, most commonly anxiety, affective disorders and substance use disorders [3]. These health compromising behaviours and mental health disorders are likely to co-occur and have the potential to adversely affect physical and mental health, and development [4].

### Opportunities for detecting health compromising behaviours and mental health disorders in primary care

Despite the high prevalence and co-occurrence of health compromising behaviours and mental health disorders, few young people seek professional help or support [5]. In Australia and worldwide, around 70 to 90 % of young people attend primary care at least once within a 12-month period [6, 7]. Whilst these consultations most commonly occur for physical complaints [7, 8], this regular attendance suggests that primary care practitioners are in the unique position to deliver opportunistic screening for mental health and health compromising behaviours as part of young people’s routine health care, providing early intervention and referrals where necessary. Indeed, more than 80 % of primary care practitioners agree that they should be responsible for identifying mental health disorders, substance abuse and behaviour problems in young people [9]. Young people also report wanting to discuss these sensitive issues with their primary care practitioner [10].

### Barriers to screening for and intervening with health compromising behaviours and mental health disorders in primary care

There are a number of major barriers to screening for and intervening with health compromising behaviours and mental health disorders in the health care setting. Practitioners report barriers to preventive care as including a lack of time to screen multiple health areas [11], feeling uncomfortable raising sensitive issues that are not the presenting issue [12], lack of awareness or skill [13], and a lack of confidence and self-efficacy in managing issues with young people [14]. Young people report concerns about embarrassment in disclosing health concerns and a lack of knowledge about available services [7, 15, 16]. The lack of private and confidential settings are also a barrier; young people who spend time alone with their primary care practitioners are more likely than those who do not to discuss sensitive topics [17].

The identification of emotional distress by primary care practitioners is highly correlated with young people recognising and expressing that they have a problem [18]. Apart from efforts to increase mental health literacy amongst young people, a screening framework that facilitates discussion of health and lifestyle is likely to increase disclosure and enhance engagement [19]. While screening for health compromising behaviours and mental health disorders do not strictly mirror screening for a disease process with a known pre-disease phase (for example dysplastic cells predating carcinoma in situ), peak health bodies recommend screening young people for health compromising behaviours and mental health disorders in primary practice [20–22]. Screening facilitates early intervention or preventive counselling efforts as young people do not often volunteer these issues as concerns to their health care providers [7].

### Available tools for screening for health compromising behaviours and mental health disorders and useful interventions

There are many screening tools for young people that focus on individual health domains, such as substance use [23], depression [24] physical activity [25] and suicide risk [26]. However, the use of tools or frameworks that enable screening of multiple health domains have a potential advantage as they address the co-occurrence of health compromising behaviours and mental health disorders in young people [27–29]. The United States and Australia have national guidelines for prevention and health promotion services to young people in primary care [21, 22]. These guidelines are age-appropriate and cover multiple and related domains, including physical health, social and academic competence, emotional wellbeing, risk reduction and violence and injury prevention. They allow primary care practitioners to normalise sensitive issues, guide discussion about behaviour and promote healthy lifestyle behaviours [30], in addition to increasing recognition of health problems that may otherwise go undetected [31].

Screening typically does not occur in isolation and, as recommended by national guidelines, should be followed up with appropriate interventions such as motivational interviewing [32], referrals to specialty care [33] or the provision of health education materials [34]. There is evidence to suggest that interventions for specific detected health issues in young people can improve depression symptoms [35], decrease weight in the obese [36] and reduce sexually transmitted infections [37]. Given these advantages, screening followed up by appropriate intervention in the primary care setting can be conceptualised as a health promotion strategy.

### Evidence for benefits of screening and intervening for multiple and commonly co-occurring health compromising behaviours and mental health disorders

Despite the potential benefits of screening for and intervening with multiple and commonly co-occurring health compromising behaviours and mental health disorders, there have been few studies that investigate the actual health impacts of interventions across multiple behavioural or risk areas. One systematic review of clinical trials by Moyer et al. [38] found that there was limited evidence to support screening and preventive interventions in young people and called for more studies in the area. A more recently published systematic review by Hale et al. [39] found that interventions for multiple risk behaviours were effective and comparable to those interventions targeting single risk factors. However, this review excluded terms for primary care and did not include young adults aged 20 to 25, who have a higher prevalence of significant health risks compared to younger adolescents [40]. Given that preventive care of young people continues to be recommended as best practice to primary care practitioners [21, 22], and central to well-care visits, it is timely to update the evidence in this setting.

This systematic review aims to investigate whether screening and subsequent interventions for multiple health compromising behaviours and mental health disorders in primary care settings improves the health outcomes of young people.

## Methods

A systematic literature review was conducted in accordance with the Preferred Reporting Items for Systematic Reviews and Meta-Analysis (PRISMA) guidelines [41] and registered on the PROSPERO website (http://www.crd.york.ac.uk/prospero/, registration number CRD42013005828). A search was conducted on 18 November, 2015 on Medline, PsycINFO, Scopus and the Cochrane Library databases using search terms representing four thematic concepts: primary care, young people, screening, and mental health and health compromising behaviour (details described in Additional file 1: Appendix 1). The reference lists of all relevant studies and reviews were also manually searched.

Individual health outcome measures were not specified in the inclusion criteria. An exhaustive search of the databases had previously been undertaken that listed individual health compromising behaviours and mental health disorders in the search terms. However this was found to be impractical as most papers returned were for individual health conditions rather than for multiple domains. Instead broader terms were tested (e.g. ‘mental disorders’ rather than ‘depression’, ‘anxiety’ ‘schizophrenia’ etc.) and papers more relevant to the objectives were found. In addition, the original intention, as stated in our Prospero Registration Form, was to exclude young people with pre-existing health conditions. However, a preliminary look at the literature returned such a small number of results that it was felt it would be reasonable to remove this exclusion criterion and in doing so acknowledge the differences in these participants in the results and discussion, including the potential limitations in generalizability.

To be eligible for inclusion studies had to: include a measure of health outcome; include at least 75 % of participants aged 25 years and under; use a screening tool or approach that assessed more than one health domain, which may have included at least one measure of behaviour modification, such as drink driving, and; be conducted within a primary care setting. As suggested by Linde et al. [42], this review included uncontrolled trials and qualitative studies to obtain an overview of the topic and inform future research. Studies were excluded if they did not evaluate health outcomes.

The first author (MW) examined all titles and abstracts extracted for relevance and read the full text of any potentially eligible articles. Articles that met the exclusion criteria were removed. The second author (SK) confirmed that all selected articles were eligible for inclusion. MW extracted data from the studies using the form described in Table 1, which was confirmed by SK. Risk of bias within each study was assessed using the Quality Rating Scale (QRS) [43]. The QRS is a 23-item instrument for the assessment of the quality of clinical trials, covering both internal validity (control of bias) and external validity (generalisability). A key advantage of the QRS over other rating scales is that it is suitable for the appraisal of non-randomized trials. Each item of the QRS is rated 0 to 2 with a total instrument range of 0 to 46; higher scores indicating better quality. The first two authors used the QRS independently. One of the potential limitations of the QRS is that it may have only moderate inter-rater agreement [43], hence inter-rater reliability was assessed by Cohen’s absolute weighted kappa statistic in Stata Version 12.0. Weighted kappa allows for different levels of agreement in ordered data, and the absolute function allows for all numbers including those unassigned by either rater.

**Table 1:** Screening tool, sample, study design, setting, intervention type, health outcomes measured, and findings of the included studies

Study	Screening tool	Sample/study design, setting	Intervention and outcomes measured	Findings	QRS^a^
Chen et al. (2011) [45]	Face-to-face (trained researchers with computer-assisted personal interviewing technology), private room within clinical setting, eligibility screen Domains screened: • substance use • sexual risk • medication adherence	*N* = 142, 16–24 years, primary care clinic for HIV positive young people, 5 sites, 45 % female, HIV positive with at least 2 of 3 HIV risk behaviours, RCT	4 × 60 minute motivational interviewing (MI) sessions focused on 2 most problematic behaviours by mental health clinicians Outcomes measured: • no condom use behaviour • risk of no condom use behaviour	Improvement: • no condom use for participants categorised as at increased sexual risk (adjusted B = .364, *p* < .01) and those categorised as not at risk (adjusted B = .325, *p* < .01) • low sexual risk (63 % vs. 32 %, *p* < .01) and likelihood to be in delayed high sexual risk group (16 % v 50 %, *p* < .01)	35.5
Mason et al. (2011) [46]	Face-to-face (trained interviewer), clinic waiting room, eligibility screen Domains screened: • substance use (incl. drink driving) • mental health	*N* = 28, 14–18 years, general primary care, 1 site, 100 % female, African American with at least 1 substance use risk, pilot RCT.	1 × 20 min MI session with a social network component by trained interviewers (not clinical staff) Outcomes measured: • substance use • trouble due to alcohol • substance use before sex • social network quality • offers to use marijuana • social stress • readiness to start counselling	Improvement: • substance use before sex (F(1) = 4.870, *p* = .038, η2 = 0.18) • social stress (F(1) = −0.187,*p* = .047, η2 = 0.16), • trouble due to alcohol use (F(1) = 4.301, *p* = .049, η2 = 0.15) • offers to use marijuana (F(1) = 4.222, *p* = .047, η2 = 0.14) No change: • substance use • social network quality • readiness to start counselling	22
Olson et al. (2008) [52]	Digital (PDA) self-administrated, waiting room, intervention screen Domains screened: • diet • exercise • screen time • substance use	11–20 years, general primary care, two cross-sectional sample recruited pre and post intervention within 5 sites and completed baseline and 6 month follow up survey. Usual care group prior to intervention: *N* = 148, 47 % female Participants recruited 1 year after intervention introduced in practices: *N* = 136, 50 % female	1 × brief MI session by trained clinician within consultation. Outcomes measured: • exercise • fruits and vegetables • milk intake • sweetened beverages • screen time	Improvement: • exercise scores between intervention (0.581) and control (−0.220, *p* = .006) • milk intake between intervention (0.190) and usual care (−0.313, *p* = .012)^b^ No change: • fruit and vegetables • sweetened beverages • screen time	23.5
Ozer et al. (2011) [51]	Pen/paper, self-administrated, waiting room, intervention screen Domains screened: • seat belt and helmet use • substance use • sexual behaviour	14 years, paediatric clinic Longitudinal study (*N* = 904) compared with several cross-sectional surveys (safety *N* = 579, sexual behaviour *N* = 1306, substance use *N* = 1410)	2 × clinical encounters: 1. provider intervention following ‘5 A’ framework for behavioural counselling; 2. health educator intervention 15–30 min informed by social cognitive theory Outcomes measured: • seat belt use • helmet use • tobacco use • alcohol use • drug use • sexual behaviour	Improvement: • helmet use (OR = 2.0, 95 %, CI = 1.1,3.7, *p* ≤ .05). No change: • smoking • alcohol • drug use • sexual behaviour	28
Patrick et al. (2006) [44]	Computer, self-administrated, immediately before intervention in the clinical office, intervention screen Domains screened: • diet • exercise	*N* = 819, 11–15 years, general primary care, 6 sites, stratified by gender (53 % female), RCT with sun exposure protection as control group. Participants booked in for a well care visit	A 12-month intervention consisting of a computer-assisted stage of readiness-based goal setting followed by brief health care provider counselling, a printed manual and 12 months of monthly mail and telephone counselling, parent intervention to help encourage change in diet and physical activity Outcomes measured: • calories from fat • fruit and vegetable servings • sedentary behaviour • minutes per week exercise • days per week exercise	Improvement: • sedentary behaviours per week for girls (% change was −12 % for intervention and 4.8 % for control group, *p* = .001) and boys (% change was −24 % for intervention and 2.4 % for control group, *p* = .001) • physical active days per week for boys (relative risk,1.47, 95 % CI: 1.19,1.75) compared to the control group No change: • calories from fat • fruit/vegetables • minutes of physical activity per week	34
Sanci et al. (2015) [48]	Practitioner (in consultation)- or self-administrated (waiting room), pen/paper, intervention screen Domains screened: • diet • exercise • substance use • mental health • violence and safety (incl. drink driving)	*N* = 901, 14–25 years, general primary care, 40 sites, 76 % female, pragmatic clustered RCT stratified by postcode advantage score and billing type	Intervention: Clinician training (9 h) in health risk screening, motivational interviewing, youth friendly practice; 2 × clinic visits. Comparison: Didactic educational seminar in youth and health risk screening Outcomes measured: • tobacco use • alcohol use • illicit drug use • risk of STI • risk of unplanned pregnancy • road safety • emotional distress	Improvement: • illicit drug use at 3 months (RD −6.0, CI:-11,−1.2; OR 0.52, CI: 0.28, 0.96) • risk for STI at 3 months (RD −5.4, CI: −11, 0.2; OR 0.66, CI: 0.46,0.96) • unplanned pregnancy at 12 months (RD −4.4; CI: −8.7, −0.1; OR 0.40, CI: 0.20,0.80) No change: • tobacco use • alcohol use • road safety • emotional distress	40
Stevens et al. (2002) [50]	Self-administrated pen/paper, subject home, intervention screen (in both intervention arms) Domains screened: • substance use • seat belt and helmet use • gun access and use	*N* = 3525^c^, paediatric clinic, 12 sites, 46 % female, 5^th^ and 6^th^ grade adolescents and parents, clustered RCT with two active arms	1 of 2 interventions: 1. home interventions (parent discussed risk with child and developed plan) plus practice intervention included MI. 2. site visits, newsletters, telephone calls; printed material Outcomes measured: • alcohol use • tobacco use • seatbelt use • helmet use • gun storage	No change: • tobacco use • seatbelt use • gun storage Negative effect: • Increased alcohol use at 24 and 36 months; OR = 1.27, 95 % CI: 1.03, 1.55, *p* = .02 and OR: 1.30, 95 % CI: 1.07, 1.57, *p* = .01, respectively	29.5
Walker et al. (2002) [47]	Face-to-face (nurse), unspecified location, intervention screen Domains screened: • mental health • physical health • substance use • diet • exercise • sexual health knowledge • health damaging behaviours	*N* = 1516, 14–16 years, general primary care, 8 sites, 51 % female, clustered RCT	1 × 20 min consultation with nurse to discuss health concerns & develop plans for healthier lifestyles based on self-efficacy and behaviour change Outcomes measured: • diet • exercise • tobacco use • alcohol use	No Change: • smoking • alcohol use • exercise • diet	26.5
Werch et al. (2007) [49]	Computer, self-administrate, immediately before intervention in quiet clinic office, intervention screen (in all 3 intervention arms) Domains screened: • exercise • diet • sleep • stress management • substance use	*N* = 155^c^, student health care, 1 site, 66 % female, 3 arms randomised trial	1 of 3 interventions from trained research staff: 1. multiple behaviour health contract based on Behavior-Image Model; 2. 1 × 25 min tailored consultation with fitness specialist; or 3. a combined consultation plus contract intervention Outcomes measured: • alcohol use • tobacco use • marijuana use • drink driving • exercise • diet • sleep • quality of life • self-control • stress management	Improvement: • drink driving behaviours in all groups (F(2136) = 4.43, *p* = .01) • exercise behaviours in all groups, (F(5140) = 6.12, *p* < .001) • nutrition habits in all groups, (F(3143) = 5.37, *p* < .001) • sleep habits in all groups (F(2144) = 5.03, *p* = .01), and health quality of life, (F(5140) = 3.09, *p* = .01) • Stress management F(2144) = 5.48, *p* = .01, and the number of health behaviour goals set in the last 30 days, F(2143) = 5.35, *p* = .01, but only among adolescents receiving the consultation, or consultation plus contract No change: • alcohol use • tobacco use • marijuana use • quality of life • self-control	25.5

^a^Average score on the Quality Rating Scale between the two raters

^b^ *t*-tests conducted on average change in health behaviours, however no statistical detail provided

^c^Age range not provided

The studies were also compared to determine whether there were similarities in the country of research, research group, or years in which the research was conducted. All studies were included irrespective of their design, quality, and biases. No statistical analyses were conducted due to the heterogeneity of study designs permitted in the inclusion criteria. Instead, we favoured narrative description of the data including statistical analyses used where appropriate.

## Results

### Summary

**Fig. 1:**
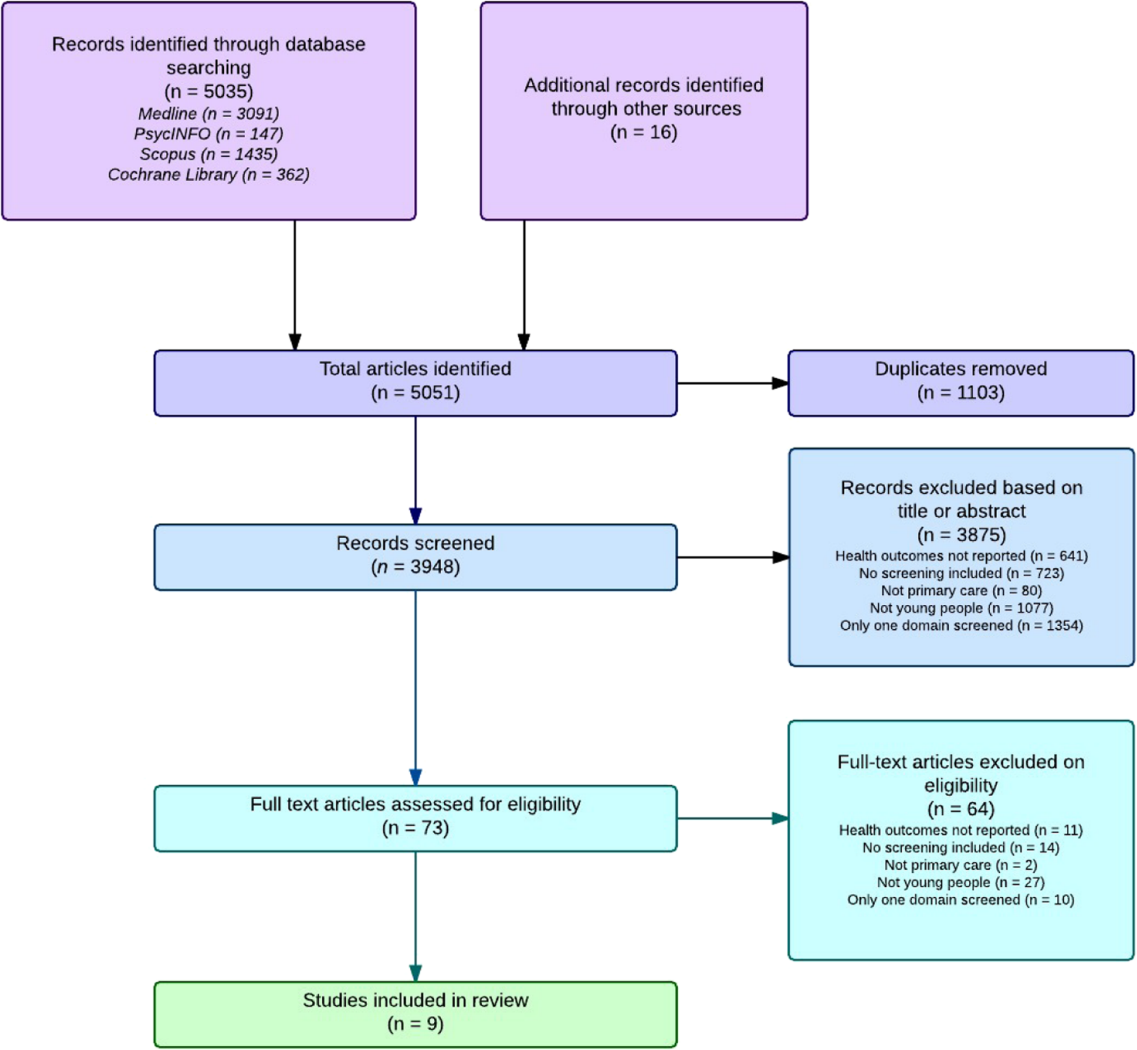
Prisma flow diagram

A total of 5051 articles were identified through the literature search, of which 1103 were excluded due to duplication, 3875 were excluded based on abstracts and a further 64 excluded after the full article length was examined, leaving a total of nine studies to review. Figure 1 depicts the PRISMA flow diagram for inclusion.

### Study characteristics

Nine publications met the inclusion criteria: Two were randomised controlled trials (RCTs) [44, 45], one was a pilot RCT [46], two were clustered RCTs [47, 48], one was a randomised study with multiple intervention groups and no control group [49], one was a cluster RCT with two active arms [50], one was a longitudinal study [51] and one was a pre-post study [52]. The majority of participants were patients at paediatric primary care [50, 51] or primary care practices [44, 46–48, 52]. Other settings were a primary care clinic at a large university [49] and primary clinics for adolescents with HIV within major metropolitan hospitals [45]. The characteristics of each study are presented in Table 1.

### Interventions

In addition to administrating a screening tool, each study included one or more interventions involving individualised motivational interviewing or counselling sessions. In seven studies [44, 47–52] the screening tool was completed as part of the intervention. The remaining two studies used the screening tool as part of the eligibility process to identify those with a risk factor who then went on to be randomised to receive the intervention or not [45, 46]. Four studies targeted individuals with increased risk of developing a disorder [47, 50–52], two targeted individuals with early signs of problems or disorder [45, 46], and three targeted all young people [44, 48, 49].

### Participants

Two studies consisted of almost all white Caucasian participants [47, 52] and five studies had a mix of Caucasian, African-American, Hispanic and/or Asian participants [44–46, 49, 51]. Stevens et al. [50] and Sanci et al. [48] did not report ethnicity details. One study was focused only on female participants [46], and the remaining seven studies involved 46–76 % of females [44, 45, 47–52]. Seven of the studies reported the mean age of participants, which was 15.6 years overall [44–47, 49–51], with the study by Olson, Gaffney, Lee, & Starr [52] reporting an age range of 11–20 years and Sanci et al. [48] reporting an age range of 14–24. Two of the nine studies examined interventions that were targeted toward at risk adolescent populations; Chen reported on effects of an intervention in a group of adolescents with HIV [52] and Mason reported on the effects of an intervention for African American adolescent females screening positive for alcohol use [53].

### Screening tools

As outlined in Table 1, participants in six of the studies self-administered the screening tools [44, 48–52], with three of these completed via a digitised system (Personal Digital Assistant [52] or computer [44, 49]). In seven studies, screening took place in a waiting room immediately before a consultation or intervention [44, 46–49, 51, 52], one was completed at an unspecified time prior to the intervention [45] and one was sent to the participants’ homes and completed by young people and their parents [50]. As a pragmatic study, Sanci et al [48] gave clinics the option of having practitioners administer the screening tool face-to-face or self-administered by young people in the waiting room. In two of the nine studies, the screening component was undertaken to test for participant eligibility for the study of the effectiveness of the intervention component [45, 46]. In the remaining seven studies the screening component was part of the intervention being tested.

Across the studies there were 11 health compromising behaviour or mental health domains used in the screening tools: substance use; mental health; physical health; diet; exercise; medication adherence; screen time; sexual health or behaviour; health damaging behaviour; sleep; and, safety (violence, gun access and use, helmet or seatbelt use). In addition, the study by Sanci et al. included screening items on home environment, activities and peer relationships, and education [48]. The screening tool of six studies had screening tools with between three and seven domains [45, 47–50, 52] and two studies had two domains [44, 46]. Substance use (tobacco, marijuana, and/or alcohol) was the most included domain [45–52], followed by exercise [44, 47–49, 52] and diet [44, 47–49, 52].

### Changes in health compromising behaviours and mental health disorders

Studies measured a range of outcomes in health compromising behaviours and mental health: diet, exercise, substance use, risky behaviour due to substance use, exercise, screen time, safety (violence, gun access and use, helmet or seatbelt use), sleep, quality of life, and sexual health or behaviour. In addition, studies also measured behaviours that may influence health compromising behaviours and mental health disorders: readiness to start counselling, social stress, and social network quality. Seven of the nine studies reported improvements in health compromising behaviours or mental health outcomes of young people in the intervention group compared to those in the control or comparison group [44–46, 48, 49, 51, 52]. Even though studies measured multiple outcomes, most studies did not report significant improvements in all outcomes measured, see Table 1. One study reported improvements in five outcomes (out of ten measured) [49], one study reported improvements in four outcomes (out of seven measured) [46], one study found improvements in three outcomes (out of seven measured) [48], three studies reported improvements in two outcomes [44, 45, 52] (out of five, two and five outcomes respectively), and one study found improvement in one outcome (out of six measured) [51].

Improvements in health outcomes were found in substance use [46, 48, 49], diet [49, 52], sexual health or risky sexual behaviour [45, 48], alcohol-related risky behaviour [46, 49], social stress [46], stress management techniques [49], helmet use [51], sleep [49], and exercise [44, 49, 52]. In addition, the study by Stevens et al. [50] reported a negative outcome; alcohol use increasing at 24 and 36 months of the intervention arm focussed on alcohol and tobacco use (OR = 1.27, 95 % CI: 1.03, 1.55, *p* = .02 and OR: 1.30, 95 % CI: 1.07, 1.57, *p* = .01, respectively). Table 1 describes study outcomes in detail.

### Assessment of bias

The QRS for both raters ranged from 20 to 40, out of a possible maximum score of 46, and had similar means. Interrater agreement was higher (95.2 %) than the expected agreement (83.3 %) with an acceptable kappa score (Rater one: M = 29.33, SD = 6.10, Rater two: M = 29.44, SD = 6.09 k =0.71, SE = 0.202, *p* = 0.0002). The average QRS for each study is listed in Table 1. The pilot RCT [46] and the pre-post study [52] scored low, between 22 and 24 for each rater, while the two RCTs [44, 45] and one of the cluster RCTs [48] scored highest, between 33 and 40 by both raters.

A number of biases were identified. There was a bias in terms of where the studies were conducted, with seven of the nine studies conducted in the United States [44–46, 49–52], one in the United Kingdom [47] and one in Australia [48]. All studies were published between 2002 and 2015, with four of the nine studies published within the last 5 years [45, 46, 48, 51]. Of the four RCT and clustered RCT studies [44, 45, 47, 48], only two reported on concealment of allocation [45, 48]. Finally, only the study by Sanci et al. [48] reported on harms or side-effects that may have occurred during the study.

### Limitations of the studies

The studies were limited by a range of methodological issues. The limitations were: a small sample size [45–47, 49, 52]; a self-selected sample, not representative and possibly biased [44, 52]; an insufficient length of follow-up time points [46, 49]; participant drop outs or non-responders [44–47, 52]; the use of potentially unreliable self-report measures [44, 45, 48]; a lack of longitudinal comparison group [51]; non-randomised design [51, 52]; multiple outcome measures carrying a greater risk of significant findings than by chance alone [46–49, 51, 52]; multiple interventions that made it difficult to determine the efficacious component [44, 48–51]; and the absence of a control condition [46, 49, 50].

## Discussion

There were nine papers included in this review, all evaluating the effect of health compromising behaviour and mental health disorder screening and interventions on young people’s health in primary care settings. Seven of these [44–46, 48, 49, 51, 52] suggested a positive change in some health outcomes, of which two were RCTs [44, 45] and one a clustered RCT [48], demonstrating reasonable support for the use of screening and intervention in primary care for young people.

A range of health compromising behaviours and mental health disorders were included in the screening component of these studies. However, only three studies screened for sexual health or behaviour [45, 48, 51], only three screened for drink driving [46, 48, 49] and only one study screened for violence [48]. As young people are at elevated risk of experiencing sexual health problems, road accidents and violence [1], there is a need to screen all young people for these issues in primary care.

Minimal information was provided about the format and administration of the screening tools, suggesting that the screening component was considered incidental or a less important component of the study by the authors. Whilst two of the studies included screening to identify individuals with health risks eligible for testing of the intervention [45, 46] we decided to retain these studies as screening was an essential component of case finding which would apply in real clinical practice should these interventions be administered. In the majority of studies [44–48, 50, 52] it was unclear if screening took place in a private space or in a waiting room where parents or others were present. Confidentiality and privacy are particularly important for young people in primary care consultations [53, 54], therefore ensuring privacy is likely to increase disclosure by young people [55].

There was also a lack of information provided about how the electronic tools [44, 49, 52] were developed and designed, and whether end-users were consulted or involved in the design of the tool’s content and functionality. Involving end-users in the design of technology ensures that the tool meets young people’s needs and is contextually relevant [56]. There is increasing interest in how technology may be incorporated into primary care to enhance service delivery and health outcomes [57] and more research is required to understand its role in screening.

With the notable exception of the study by Sanci et al. [48], the samples included in this review were under 20 years of age [45, 46, 50–52] and most studies were conducted in the United States [44, 46, 47, 49–52] limiting generalisability of this review for other countries and health systems. As most studies included younger adolescents, the extent to which the results of this review can be extended to young adults aged 18 to 25 is unknown. Young adults have a higher prevalence of significant health risks compared to younger adolescents aged 12–17 and adults aged 26–34 [40]. This young adult population is also more likely to report not having a regular doctor and having a weaker sense of belonging to a local community, likely due to being socially isolated and to stressors from work and study commitments, compared to other age groups [58].

Only two studies reported on harms or negative effects that may have occurred during the study. Harms may include the additional costs and medical procedures associated with false-positive screening results, or a paradoxical increase in an unhealthy behaviour [38]. The study by Sanci et al. [48] reported no harmful events, while Stevens et al. [50] reported a negative outcome effect of increased alcohol use in the intervention arm focussed on alcohol and tobacco use. The Dartmouth Prevention Project reported by Stevens differed from the other studies in that included subjects were pre-adolescents in the fifth or sixth grade and their parents, and the intervention rested on encouraging communication between parents and children about health risks rather than the clinician communicating directly with an adolescent. Authors proffer that the observed results may have arisen from family discussions that may have been broader ranging than the topics in focus or that the intervention was insufficient to curb short term alcohol use; without follow-up into later adolescence when uptake of alcohol is greater, full effect on preventing use could not be evaluated. Future research needs to explicitly measure potential harms or risks in screening for and intervening with multiple health compromising behaviours and mental health disorders.

Limitations in the study designs may have minimised the generalisability of the findings. Three of the seven studies that detected a change in health compromising behaviour or mental health scored high in the QRS; two RCTs [44, 45] and one clustered RCT [48]. However, one of these RCTs was targeted at HIV positive young people [45], so results may not be replicable in a general population of young people attending primary care. In addition, of the four remaining studies that found improvements in health compromising behaviour or mental health, three had the lowest QRS scores of the nine studies: a pilot RCT [46], a pre-post design study [52] and a randomised trial with no control [49]. All of these studies had small sample sizes and high participant drop outs, while the study by Mason et al. [46] was targeted at urban African females, and both Mason et al. [46] and Werch et al. [49] had only 1 month follow ups.

Other limitations in study designs may have minimised the ability to detect a change in health compromising behaviour or mental health outcomes. For instance, Walker et al. [47] had small participant numbers in each domain that was measured, as well as high rates of non-response, particularly at 12 months, which may account for the lack of effectiveness observed in this study. Other important potential limitations are the challenges inherent in measuring changes in multiple health outcomes. It is difficult to obtain sufficient statistical power to show shifts in several outcomes and measuring multiple outcomes increases the potential for positive findings by chance alone [38]. It is hard to overcome this latter challenge without a combined risk variable, which also has drawbacks in that detail of which risks are affected and which are not is lost. It is noteworthy that not all outcomes targeted in these interventions changed; we did not view this negatively as it is highly unlikely a young person with co-occurring health risks will modify all behaviours simultaneously. Studies require longer follow-up times to test whether interventions have cumulative effects with subsequent exposures over time [55].

It is interesting to note that none of the studies assessed changes for known protective factors for young people’s health, such as school engagement, employment and meaningful relationships [1, 59]. For example, family and school connectedness protect against emotional distress, suicidal thoughts and behaviours, violence, substance use and early age of initial sexual experience [13]. Conversely, there is an increased risk of dropping out of education associated with cannabis use, suicide attempts, depression and welfare dependence [60]. Identifying and discussing protective factors with young people is central to the strength-based approach which promotes healthy development and the reduction of health compromising behaviours [61]. The strength-based approach is recommended by national guidelines for the prevention and health promotion services to young people in primary care [21, 22]. More research is needed to investigate the effectiveness of adolescent screening and intervention in primary practice which includes the identification and discussion of both health compromising and protective factors.

Several national guidelines recommend the use of screening tools and intervention with young people [21, 22]. However, while this review demonstrates that there is limited high quality evidence that screening across multiple health risks and intervention in primary care improves the mental health or health compromising behaviours of young people, there is evidence of modest impact with both high and lower quality studies showing positive shifts in some of the health domains they targeted. Is this sufficient to support the guideline recommendations? Some argue that sample sizes required to counter the challenges in measuring multiple outcomes from screening and associated interventions preclude trial methodologies and rather commend efficacy studies after widespread implementation [62]. Follow-up times are seldom long enough in trials to assess whether outcomes that take years to develop are prevented [38]. In addition the psycho-social risks that burden adolescents’ health have complex aetiologies and require multifaceted interventions including school, peers, families and community, not just clinical settings [63]. Economic evaluations of adolescent interventions are also lacking hence making it difficult to compare interventions on cost versus benefits [63]. Hence it remains difficult to provide strong evidence for the relative benefits of guideline recommended screening and intervention in clinical settings alone versus other types of intervention for detecting, preventing and reducing adolescents’ health risks. Future research is needed to determine more effective methods for assisting clinicians to identify mental health disorder or health compromising behaviour in young people, or for assisting vulnerable young people to self-identify and seek appropriate help. Testing these clinical interventions as part of a multifaceted approach to health risks is also warranted. In addition work remains to review which interventions are most effective to tackle particular health risks in young people as case identification alone is unlikely to help [38, 64].

## Limitations

This review has a number of limitations. Firstly, while including uncontrolled studies allowed for a broad overview of research in this area to date, this meant that a meta-analysis was not possible. Another limitation was that as this review investigated studies that combined screening and at least one intervention it is possible that eligible articles that were not categorised with the theme of ‘screening’ as a key term in the databases were not picked up in the search.

## Conclusions

This systematic review suggests that there is some evidence that the use of screening and intervention with young people for mental health disorder or health compromising behaviours in clinical settings improves health outcomes. Given that young people have expressed a desire and willingness to engage in these discussions and trust clinicians’ advice, this review suggests that these discussions should be part of the routine primary care of young people. There is a need for further quality studies, able to overcome the challenges of measuring change in multiple outcomes over time, to strengthen this evidence. Study quality might be enhanced by following young people over time to capture return visits to clinicians and effects of these repeat exposures to screening and intervention on health outcomes, and by conducting a trial alongside wide scale implementation in a population with greater numbers of young people and more study power. Further work might also examine the role of this clinical intervention in multifaceted approaches across health, education and community settings to tackle complex adolescent risky behaviour. This review also highlights a need for research including young adults (18–25 years) and research of screening that includes the identification of protective factors.

## Additional file

Additional file 1: Appendix 1. Terms used in the literature search. Terms used in the literature search for each database. (DOCX 17 kb)

## References

[1] Sawyer SM, Afifi RA, Bearinger LH, Blakemore S-J, Dick B, Ezeh AC, Patton GC (2012). Adolescence: a foundation for future health. Lancet.

[2] Gore FM, Bloem PJN, Patton GC, Ferguson J, Joseph V, Coffey C, Sawyer SM, Mathers CD (2011). Global burden of disease in young people aged 10–24 years: a systematic analysis. Lancet.

[3] Kessler RC, Berglund P, Demler O, Jin R, Merikangas KR, Walters EE (2005). Lifetime prevalence and age-of-onset distributions of DSM-IV disorders in the national comorbidity survey replication. Arch Gen Psychiatry.

[4] Spring B, Moller AC, Coons MJ (2012). Multiple health behaviours: overview and implications. J Public Health (Bangkok).

[5] Anderson JE, Lowen CA (2010). Systematic review: connecting youth with health services. Can Fam Physician.

[6] Australian Bureau of Statistics. Australian Health Survey: Health Service Usage and Health Related Actions, 2011-12. Canberra: ABS; 2013.

[7] Tylee A, Haller DM, Graham T, Churchill R, Sanci L (2007). Youth-friendly primary-care services: how are we doing and what more needs to be done?. Lancet.

[8] Booth ML, Knox S, Kang M (2008). Encounters between adolescents and general practice in Australia. J Paediatr Child Health.

[9] Stein REK, Horwitz SM, Storfer-isser A, Heneghan A, Olson L, Hoagwood KE (2008). Do pediatricians think they are responsible for identification and management of child mental health problems? Results of the AAP periodic survey. Ambul Pediatr.

[10] Klein JD, Wilson KM (2002). Delivering quality care: adolescents’ discussion of health risks with their providers. J Adolesc Heal.

[11] Yarnall KSH, Pollak KI, Østbye T, Krause KM, Michener JL (2003). Primary care: is there enough time for prevention?. Am J Public Health.

[12] Henry-Reid LM, O’Connor KG, Klein JD, Cooper E, Flynn P, Futterman DC (2010). Current pediatrician practices in identifying high-risk behaviors of adolescents. Pediatrics.

[13] Chung PJ, Lee TC, Morrison JL, Schuster MA (2006). Preventive care for children in the United States: quality and barriers. Annu Rev Public Health.

[14] Jarrett C, Dadich A, Robards F, Bennett D (2011). “Adolescence is difficult, some kids are difficult”: general practitioner perceptions of working with young people. Aust J Prim Health.

[15] Booth ML, Bernard D, Quine S, Kang MS, Usherwood T, Alperstein G, Bennett DL (2004). Access to health care among Australian adolescents young people’s perspectives and their sociodemographic distribution. J Adolesc Heal.

[16] Bernard D, Quine S, Kang M, Alperstein G, Usherwood T, Bennett D, Booth M (2004). Access to primary health care for Australian adolescents: how congruent are the perspectives of health service providers and young people, and does it matter?. Aust N Z J Public Health.

[17] Fairbrother G, Scheinmann R, Osthimer B, Dutton MJ, Newell K-A, Fuld J, Klein JD (2005). Factors that influence adolescent reports of counseling by physicians on risky behavior. J Adolesc Health.

[18] Haller DM, Sanci LA, Sawyer SM, Patton GC (2009). The identification of young people’s emotional distress: a study in primary care. Br J Gen Pract.

[19] Bradford S, Rickwood D (2012). Psychosocial assessments for young people: a systematic review examining acceptability, disclosure and engagement, and predictive utility. Adolesc Health Med Ther.

[20] Department of Health (UK) (2011). Quality Criteria for Young People Friendly Health Services.

[21] American Academy of Pediatrics. Bright Futures: Guidelines for Health Supervision of Infants, Children, and Adolescents. Elk Grove Villange, IL: American Academy of Pediatrics; 2008.

[22] Royal Australian College of General Practitioners (2012). Guidelines for Preventive Activities in General Practice.

[23] Gryczynski J, Kelly SM, Mitchell SG, Kirk A, O’Grady KE, Schwartz RP (2015). Validation and performance of the Alcohol, Smoking and Substance Involvement Screening Test (ASSIST) among adolescent primary care patients. Addiction.

[24] Allgaier A-K, Pietsch K, Fruehe B, Sigl-Gloeckner J, Schulte-Koerne G (2012). Screening for depression in adolescents: validity of the Patient Health Questionnaire in pediatric care. Depress Anxiety.

[25] Ball TJ, Joy EA, Goh TL, Hannon JC, Gren LH, Shaw JM (2015). Validity of two brief primary care physical activity questionnaires with accelerometry in clinic staff. Prim Health Care Res Dev.

[26] Horowitz LM, Bridge JA, Teach SJ, Ballard E, Klima J, Rosenstein DL, Wharff EA, Ginnis K, Cannon E, Joshi P, Pao M (2012). Ask Suicide-Screening Questions (ASQ): a brief instrument for the pediatric emergency department. Arch Pediatr Adolesc Med.

[27] Brener ND, Collins JL (1998). Co-occurrence of health-risk behaviors among adolescents in the United States. J Adolesc Heal.

[28] Burnett-Zeigler I, Walton MA, Ilgen M, Barry KL, Chermack ST, Zucker RA, Zimmerman MA, Booth BM, Blow FC (2012). Prevalence and correlates of mental health problems and treatment among adolescents seen in primary care. J Adolesc Heal.

[29] Hair EC, Park MJ, Ling TJ, Moore K. Risky behaviors in late adolescence: co-occurrence, predictors, and consequences. J. Adolesc. Health. 2009;45:253–61 . 10.1016/j.jadohealth.2009.02.009 19699421

[30] Fothergill KE, Gadomski A, Solomon BS, Olson AL, Gaffney CA, DosReis S, Wissow LS (2013). Assessing the impact of a web-based comprehensive somatic and mental health screening tool in pediatric primary care. Acad Pediatr.

[31] Paul C, Yoong SL, Sanson-Fisher R, Carey M, Russell G, Makeham M (2014). Under the radar: a cross-sectional study of the challenge of identifying at-risk alcohol consumption in the general practice setting. BMC Fam Pract.

[32] Rubak S, Sandbaek A, Lauritzen T, Christensen B (2005). Motivational interviewing: a systematic review and meta-analysis. Br J Gen Pract.

[33] Husky MM, Miller K, McGuire L, Flynn L, Olfson M (2010). Mental health screening of adolescents in pediatric practice. J Behav Health Serv Res.

[34] Klein JD, Allan MJ, Elster AB, Stevens D, Cox C, Hedberg VA, Goodman RA (2001). Improving adolescent preventative care in community health centers. Pediatrics.

[35] Williams S, O’Connor E, Whitlock E. Screening for child and adolescent depression in primary care settings: a systematic evidence review for the U.S. Preventive Services Task Force. Rockville: Agency for Healthcare Research and Quality; 2009. 20722167

[36] US Preventive Services Task Force (2010). Screening for obesity in children and adolescents: US Preventive Services Task Force recommendation statement. Pediatrics.

[37] Lin JS, Whitlock E, Connor EO, Bauer V (2008). Behavioral counseling to prevent sexually transmitted infections: a systematic review for the U.S. Preventive Services Task Force. Ann Intern Med.

[38] Moyer V, Butler M (2004). Gaps in the evidence for well-child care: a challenge to our profession. Pediatrics.

[39] Hale DR, Fitzgerald-Yau N, Viner RM (2014). A systematic review of effective interventions for reducing multiple health risk behaviors in adolescence. Am J Public Health.

[40] Neinstein LS. The new adolescents: an analysis of conditions, behaviors, risks, and access to services among emerging young adults. Volume 208 Suppl. Los Angeles: University of Southern California; 2013.

[41] Moher D, Liberati A, Tetzlaff J, Altman DG (2009). Preferred reporting items for systematic reviews and meta-analyses: the PRISMA statement. Ann Intern Med.

[42] Linde K, Scholz M, Melchart D, Willich SN (2002). Should systematic reviews include non-randomized and uncontrolled studies? The case of acupuncture for chronic headache. J Clin Epidemiol.

[43] Moncrieff J, Churchill R, Drummond DC, Mcguire H (2006). Development of a quality assessment instrument for trials of treatments for depression and neurosis. Int J Methods Psychiatr Res.

[44] Patrick K, Calfas KJ, Norman GJ, Zabinski MF, Sallis JF, Rupp J, Covin J, Cella J (2006). Randomized controlled trial of a primary care and home-based intervention for physical activity and nutrition behaviors. Arch Pediatr Adolesc Med.

[45] Chen X, Murphy DA, Naar-King S, Parsons JT (2011). A clinic-based motivational intervention improves condom use among subgroups of youth living with HIV. J Adolesc Heal.

[46] Mason M, Pate P, Drapkin M, Sozinho K (2011). Motivational interviewing integrated with social network counseling for female adolescents: a randomized pilot study in urban primary care. J Subst Abuse Treat.

[47] Walker Z, Townsend J, Oakley L, Donovan C, Smith H, Bell J, Marshall S, Hurst Z, Surgery M (2002). Health promotion for adolescents in primary care: randomised controlled trial. BMJ.

[48] Sanci L, Chondros P, Sawyer S, Pirkis J, Ozer E, Hegarty K, Yang F, Grabsch B, Shiell A, Cahill H, Ambresin A-E, Patterson E, Patton G (2015). Responding to young people’s health risks in primary care: a cluster randomised trial of training clinicians in screening and motivational interviewing. PLoS One.

[49] Werch CEC, Bian H, Moore MJ, Ames S, DiClemente CC, Weiler RM (2007). Brief multiple behavior interventions in a college student health care clinic. J Adolesc Heal.

[50] Stevens M, Olson A, Gaffney C, Tosteson T, Mott L, Starr P (2002). A pediatric, practice-based, randomized trial of drinking and smoking prevention and bicycle helmet, gun, and seatbelt safety promotion. Pediatrics.

[51] Ozer E, Adams SH, Orrell-Valente JK, Wibbelsman CJ, Lustig JL, Millstein SG, Garber AK, Irwin CE (2011). Does delivering preventive services in primary care reduce adolescent risky behavior?. J Adolesc Heal.

[52] Olson AL, Gaffney CA, Lee PW, Starr P (2008). Changing adolescent health behaviors: the healthy teens counseling approach. Am J Prev Med.

[53] Coker TR, Sareen HG, Chung PJ, Kennedy DP, Weidmer BA, Schuster MA (2010). Improving access to and utilization of adolescent preventive health care: the perspectives of adolescents and parents. J Adolesc Heal.

[54] Ambresin A-E, Bennett K, Patton GC, Sanci LA, Sawyer SM (2013). Assessment of youth-friendly health care: a systematic review of indicators drawn from young people’s perspectives. J Adolesc Heal.

[55] Wissow LS, Brown J, Fothergill KE, Gadomski A, Hacker K, Salmon P, Zelkowitz R (2013). Universal mental health screening in pediatric primary care: a systematic review. J Am Acad Child Adolesc Psychiatry.

[56] Clemensen J, Larsen SB, Kyng M, Kirkevold M (2007). Participatory design in health sciences: using cooperative experimental methods in developing health services and computer technology. Qual Health Res.

[57] Dadich A, Jarrett C, Sanci L, Kang M, Bennett D (2013). The promise of primary health reform for youth health. J Paediatr Child Health.

[58] Marshall EG (2011). Do young adults have unmet healthcare needs?. J Adolesc Heal.

[59] Resnick MD, Bearman PS, Blum RW, Bauman KE, Harris KM, Jones J, Tabor J, Beuhring T, Sieving RE, Shew M, Ireland M, Bearinger LH, Udry JR (1997). Protecting adolescents from harm: findings from the national longitudinal study on adolescent health. JAMA.

[60] Silins E, Horwood LJ, Patton GC, Fergusson DM, Olsson CA, Hutchinson DM, Spry E, Toumbourou JW, Degenhardt L, Swift W, Coffey C, Tait RJ, Letcher P, Copeland J, Mattick RP (2014). Young adult sequelae of adolescent cannabis use: an integrative analysis. The Lancet Psychiatry.

[61] Duncan PM, Garcia AC, Frankowski BL, Carey PA, Kallock EA, Dixon RD, Shaw JS (2007). Inspiring healthy adolescent choices: a rationale for and guide to strength promotion in primary care. J Adolesc Heal.

[62] Downs SM, Klein JD (1995). Clinical preventive services efficacy and adolescents’ risky behaviors. Arch Pediatr Adolesc Med.

[63] Sanci L (2011). Clinical preventive services for adolescents: facing the challenge of proving “an ounce of prevention is worth a pound of cure”. J Adolesc Heal.

[64] Sanci L, Lewis D, Patton G (2010). Detecting emotional disorder in young people in primary care. Curr Opin Psychiatry.

