# Supplementary material for: Does screening for and intervening with multiple health compromising behaviours and mental health disorders amongst young people attending primary care improve health outcomes? A systematic review
Source: BMC Fam Pract. 2016 Aug 4;17:104. doi: 10.1186/s12875-016-0504-1 (PMC4973106; doi:10.1186/s12875-016-0504-1)
Supplement: Additional file 1: — Appendix 1. Terms used in the literature search. Terms used in the literature search for each database. (DOCX 17 kb) [file 12875_2016_504_MOESM1_ESM.docx]

**Appendix 1: Terms used in the literature search**

## SEARCH 1 & 2: Medline and the Cochrane Review (conducted 18/11/15)

MeSH terms and Topic Field were searched as indicated by ^M^ or ^T^ respectively. No limits were set in this search.

|  | 1. Primary Health Care^M^ OR General Practice^M^ OR Family Practice^M^ OR "primary care"^T^ OR "primary practice"^T^ OR "family medicine"^T^   AND |
| --- | --- |

1. Mass Screening^M^ OR Self-Assessment^M^ OR Risk Assessment^M^ OR Needs Assessment^M^ OR screen*^T^ OR assess*^T^

AND

1. Adolescent^M^  OR Young Adult^M^ OR  teen*^T^ OR “young person”^T^ OR youth^T^

AND

1. Mental Disorders^M^ OR Mental Health^M^ OR Risk Factors^M^ OR Risk-Taking^M^ OR “protective factors”^T^ OR wellbeing^T^ OR psychosocial*^T^ OR multidimensional^T^

## Search 3: PsycINFO (conducted 18/11/15)

Thesaurus terms and Subject terms were searched as indicated by ^DE^ and ^SU^ respectively. No limits were set in this search.

1. "Primary Health Care"^DE^ OR "Family Medicine"^DE^ OR "general practice"^SU^ OR "family practice"^SU^ OR "primary care"^SU^ OR "primary practice"^SU^

AND

1. "Adolescent Development"^DE^ OR teen*^SU^ OR "young person"^SU^ OR youth^SU^ OR "young adult"^SU^ OR adolescent^SU^

AND

1. "Health Screening"^DE^ OR (DE "Risk Assessment"^DE^ OR "Needs Assessment"^DE^ OR "self assessment"^SU^ OR screen*^SU^ OR assess*^SU^

AND

1. "Mental Disorders"^DE^ OR "Mental Health"^DE^ OR "Risk Factors"^DE^ OR "Risk Taking"^DE^ OR "Protective Factors"^DE^ OR "Psychosocial Factors"^DE^ OR wellbeing^SU^ OR multidimensional^SU^

## Search 4: Scopus (conducted 18/11/15)

Keyword search used for all terms. No limits were set in this search.

1. "Primary Health Care"  OR  "Family Medicine"  OR  "general practice"  OR  "family practice"  OR  "primary care"  OR  "primary practice"

AND

1. "Health Screening"  OR  "Risk Assessment"  OR  "Needs Assessment"  OR  screen*  OR  assess*

AND

1. "Young Adult"  OR  adolescent  OR  teen*  OR  "young person"  OR  youth

AND

1. "Mental Disorders"  OR  "Mental Health"  OR  "Risk Factors"  OR  "Risk-taking*"  OR  "protective factors"  OR  wellbeing  OR  psychosocial  OR  multidimensional
